# Nutrition economic evaluation of allergy treatment in infants and children: background for probiotic studies

**DOI:** 10.3402/mehd.v23i0.18584

**Published:** 2012-06-18

**Authors:** Soili Alanne

**Affiliations:** 1 Department of Clinical Nutrition, Seinäjoki Central Hospital, Hospital District of South Osthrobothnia, Seinäjoki; 2 Functional Foods Forum, University of Turku, Finland

**Keywords:** food allergy, atopic eczema, children, nutrition, probiotic, economics

## Abstract

The treatment of food allergy is based on avoidance of the foods, which cause symptoms, and their replacement with nutritionally comparable foods. The cost of food allergy and elimination diets to families and society is poorly known. Our results suggest that estimation of dietary costs on the basis of dietary records was possible but challenging. In infancy, cost differences were small but vary depending on the age group with the reduction of median yearly costs around 180–240€. Thus, further studies are required for a more accurate cost estimate and an estimation of the impact of specific probiotics.

In infancy and early childhood, a balanced diet is essential to ensure age-appropriate growth and development. Dietary guidelines recommend exclusive breastfeeding until the age of 6 months and introduction of supplemental foods before the age of 6 months (1). In infants with food allergy, the introduction of supplemental foods is often delayed and the variety of foods restricted due to allergic symptoms. Mothers often feel unsuccessful when they cannot proceed according to suggested feeding guidelines (2). The organ systems most commonly involved in food allergy include the skin, gastrointestinal tract, and respiratory tract (3). Allergic reactions to food have been reported in 71% of children with severe eczema and in 51% of children who had less severe eczema initially (4). In Europe, parents perceive that 7.2% of children aged 2–3 years have food allergy (5), and diagnosed food allergy affects 5–6% of children by the age of 3 years based on double-blinded, placebo-controlled food challenge and good clinical history (6). The treatment of food allergy is based on avoidance of the foods that have been identified as allergens and their substitution with nutritionally comparable foods. The cost of food allergy and elimination of diets to families and society is poorly known. Knowledge about diet and treatment costs is needed for a basis of economic evaluations. In regard to pre- and probiotics in the treatment or prevention of eczema and food allergy, the costs of probiotic preparation should be compared against costs caused by a disease.

When two or more treatment alternatives are compared, but in which the costs and consequences of each are not examined, the terms are either efficacy and effectiveness evaluations or cost analyses. These are only partial evaluations; however, full economic evaluation includes usually both. Analyses, in which costs are related to a single, common effect that may differ in magnitude between the alternative programmes are referred to as cost-effectiveness analyses. The results may be stated as cost per unit of effect. In cost-utility analyses, utility refers to the preferences of individuals or society. The generic outcome as expressed by quality-adjusted life year (QALY) is arrived at in each case by adjusting the length of time affected through the health outcome by the utility value on a scale of 0–1 of the resulting level of health status (7).

Previous studies have evaluated the financial cost of childhood eczema, including dietary costs in Australian families. The additional annual dietary cost was 0 AUD in mild, 81 AUD (∼51€ in 1997) in moderate, and 360 AUD (∼227€ in 1997) in severe eczema (8). Data were collected by a questionnaire covering 12 months, but no details were given about these costs. An Italian study (9) focused also on eczema, where families were assessed by questionnaire about the use of food products with information about the name of the product, the cost, and quantity used in 1 month. The annual cost of these food products were 117 and 452€ of family costs in moderate and severe eczema, respectively. Thirty percent of all children used food products, mainly formulas for cow's milk allergic children, with an increase of 684€ to standard diet (9). The three most often reported cost components to families of children with eczema include hospitalization, home environmental changes, medical consultations, time off work, over the counter medicines, medications, and moisturizers (8–11).

Preference weights that are needed for a basis of cost-effectiveness analysis have been traced for different severity of eczema. Mean preference scores for mild, mild to moderate, moderate, moderate to severe, and severe eczema were 91, 84, 73, 61, and 49, respectively, in a scale of 100 (=1) (perfect health) to 0 (death) (12). In another study, the valuation survey of parents of children with eczema and general population estimated preference weights for each of 16 different health states value ranging from 0.36 to 0.84 on a continuum of 0 (death) to 1 (13). Studies related to food allergy and cow's milk allergy have been mainly decision analyses evaluations from different perspectives like health care in from the perspective of healthcare insurers (14, 15), publicly funded healthcare systems (16, 17), and parents of infants (14). In these analyses, major costs to public health care have accrued from clinician and GP visits (44–50% of costs) and nutrition preparations (38–87% of costs). Little is thus known about dietary costs.

This study was designed to evaluate challenges related to estimation of daily dietary costs and factors contributing to these costs in infants with and without food allergy at the ages of 6, 12, and 24 months and challenges related to evaluation of food-related costs longitudinally.

## Subjects and methods

Children (*N*=80, 60.3% boys) with (*n*=23) and without (*n*=57) food allergy were evaluated at the ages of 6, 12, and 24 months. Nutrient intake and diet-related costs were calculated from 3-day diet records. Food prices were obtained from local supermarkets and prices of vitamin and mineral supplements and infant formula from the University Pharmacy. Growth, length of breastfeeding, and age at introduction of solid foods were ascertained. Data on reimbursements for hydrolyzed formulas used by infants with cow's milk allergy were obtained from the Social Insurance Institution of Finland. Correlations between average daily costs and other factors were analyzed, and paired *t*-tests were used to analyse differences in costs. Prices of year 2006 were used.

## Results

There were differences in energy yielding nutrient intakes between the infants with and without food allergy. Protein intake was lower (*p*=0.001) and fat intake was (*p*=0.04) higher in infants with food allergy at the ages of 12 and 24 months (18). The daily dietary costs for families with infants with food allergy were 1.64€ (SD 1.62, *n*=21), 3.18€ (SD 1.5, *n*=23), and 2.91€ (SD 0.82, *n*=20) and without food allergy 1.21€ (SD 0.94, *n*=55), 2.69€ (SD 0.7, *n*=53), and 2.89€ (SD 0.61, *n*=55) at the ages of 6, 12, and 24 months, respectively. However, the costs were not significantly higher than in infants without food allergy (12 months, *p*=0.146; 24 months, *p*=0.915). The infants with food allergy who used hydrolyzed formula (*n*=12) had a higher daily dietary cost than those using (*n*=11) soy-, oat-, or rice-based alternatives or breastfed at 12 months (3.91€ vs. 2.41€, *p*=0.015). Society's mean contribution (Social Insurance Institution) to the cost of using hydrolyzed formula was 8.67€ (SD 7.78) and 4.86 (SD 5.15) per child at the ages of 12 and 24 months, respectively, which means that the cost to families would be much higher without a reimbursement. Daily use of probiotic preparation would add daily dietary cost by 0.5–0.7€ (2012 price) per day. Longer breastfeeding was related to lower dietary costs in infants with and without food allergy at 12 months (*r*= − 0.58, *p*=0.004; *r*= − 0.37, *p*=0.006).

## Discussion

Nutrient intakes have been reported in studies earlier, but food-related costs have been seldom evaluated. In infancy, this is challenging when the age of introduction of infant formula or complementary feeding varies. Management of infant's food allergy by elimination diet only modestly increased the family's daily dietary costs. Provided that specific probiotics or prebiotics are effective to alleviating the symptoms like eczema, previous case scenarios and measured preference weights may serve a basis for calculation of QALY. This, however, means that the severity of eczema is measured at more frequent intervals with reliable and validated measures of severity and own specific measure for those having gastrointestinal symptoms, for example visual analogue scale, to identify the differences resulting from probiotic or prebiotic use. Thus, from the economic perspective, the reduction of median yearly costs should be 180–240€ (2012 value), which means a shift from severe to moderate or moderate to mild eczema. When the costs of probiotic or prebiotic use are estimated, the total reduction in costs can be evaluated either against this common measure or the number of prevented cases.

## Conclusion

The estimation of dietary costs on the basis of dietary records was possible but challenging. In infancy, cost differences were quite small. The diet is put into practice in different ways even in infants with cow's milk allergy. Introduction of solid foods and breastfeeding were related to lower dietary costs, but otherwise mothers choices when and how to start complementary feeding seems to be more relevant. In those infants with more severe allergy, hydrolyzed formulas are the main reason for increased costs. Daily average costs can be used to estimate yearly costs because more accurate data are difficult to obtain. It would require longitudinal dietary records over months, which are usually impossible to obtain.
